# Improved Drug-Response Prediction Model of APC Mutant Colon Cancer Patient-Derived Organoids for Precision Medicine

**DOI:** 10.3390/cancers15235531

**Published:** 2023-11-22

**Authors:** Yong Jae Shin, Eun Hae Jo, Yunjeong Oh, Da Som Kim, Seungyoon Hyun, Ahran Yu, Hye Kyung Hong, Yong Beom Cho

**Affiliations:** 1 Innovative Institute for Precision Medicine, Samsung Medical Center, Seoul 06351, Republic of Korea; yongjae.shin@samsung.com (Y.J.S.); eunhaej@andrew.cmu.edu (E.H.J.); yunjeong.oh@sbri.co.kr (Y.O.); dasom21.kim@sbri.co.kr (D.S.K.); hyekyung.hong@samsung.com (H.K.H.); 2 Department of Surgery, Samsung Medical Center, Sungkyunkwan University School of Medicine, Seoul 06351, Republic of Korea; yu.ahran@gmail.com; 3 Department of Health Sciences and Technology, Samsung Advanced Institute for Health Science & Technology (SAIHST), Sungkyunkwan University, Seoul 06351, Republic of Korea; soonna1005@gmail.com; 4 Department of Biopharmaceutical Convergence, Sungkyunkwan University, Suwon-si 16419, Republic of Korea

**Keywords:** colorectal cancer, patient-derived organoids (PDOs), oxaliplatin, precision medicine, drug response prediction, first-line chemotherapy, FLOFIRI/FOLFOX, adenomatous polyposis coli (APC)

## Abstract

**Simple Summary:**

As a first-line chemotherapeutic for colorectal cancer (CRC), FOLFOX, XELOX, and similar regimens are recommended as adjuvant therapies following CRC surgery, according to the NCCN guidelines. In stage IV patients, targeted therapies like cetuximab and bevacizumab are used in combination. The decision to add either irinotecan-based regimens or oxaliplatin-based regimens, FOLFIRI is typically based on a physician’s subjective judgment due to the lack of clear selection criteria. Therefore, having the ability to precisely predict the therapeutic response to these two drugs could help prioritize the use of the most effective drug in stage IV patients. Previous research efforts aimed to predict therapeutic responses to these two drugs using organoids. While irinotecan showed promising predictability, predicting the response to oxaliplatin remained challenging. In this study, new drug screening conditions were identified to address this issue. Through additional research using CRC organoids, this paper contributes to the prediction of drug responses in CRC patients.

**Abstract:**

Colorectal cancer is the third most common cancer in the world, with an annual incidence of 2 million cases. The success of first-line chemotherapy plays a crucial role in determining the disease outcome. Therefore, there is an increasing demand for precision medicine to predict drug responses and optimize chemotherapy in order to increase patient survival and reduce the related side effects. Patient-derived organoids have become a popular in vitro screening model for drug-response prediction for precision medicine. However, there is no established correlation between oxaliplatin and drug-response prediction. Here, we suggest that organoid culture conditions can increase resistance to oxaliplatin during drug screening, and we developed a modified medium condition to address this issue. Notably, while previous studies have shown that survivin is a mechanism for drug resistance, our study observed consistent survivin expression irrespective of the culture conditions and oxaliplatin treatment. However, clusterin induced apoptosis inhibition and cell survival, demonstrating a significant correlation with drug resistance. This study’s findings are expected to contribute to increasing the accuracy of drug-response prediction in patient-derived APC mutant colorectal cancer organoids, thereby providing reliable precision medicine and improving patient survival rates.

## 1. Introduction

Colorectal cancer (CRC) is a malignant tumor that originates in the colon and rectum. It is the second most commonly occurring cancer in the United States, with approximately 150,000 patients diagnosed with CRC annually. Approximately 90% of CRC cases can be detected by regular screening, but it remains the second leading cause of cancer deaths overall [1,2,3,4].

**Figure 1:**
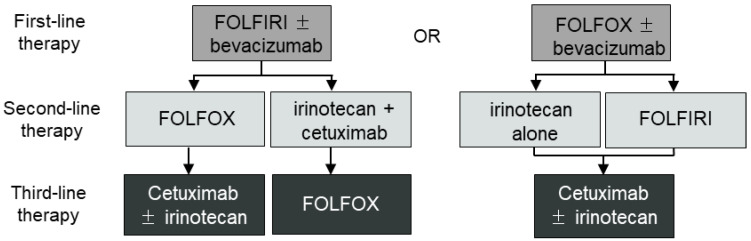
Colorectal cancer first-line therapy guideline. Treatment options for metastatic colorectal cancer patients. Cetuximab or panitumumab for patients with Kras wild-type tumors. FOLFIRI = irinotecan plus infusional 5-fluorouracil/leucovorin; FOLFOX = oxaliplatin plus infusional 5-fluorouracil/leucovorin.

Previously, 5-fluorouracil (5-FU) was the only treatment option available, and the average survival time was approximately 12 months [5]. Nevertheless, according to the reports indicating that the use of various combination chemotherapy regimens can extend the survival time, FOLFIRI (5-FU/leucovorin/irinotecan) and FOLFOX (5-FU/leucovorin/oxaliplatin), which enhance the effectiveness of 5-FU with leucovorin, irinotecan, or oxaliplatin, are commonly used as the first-line treatment (Figure 1) [6,7,8,9]. Following the NCCN guidelines, 5-FU is commonly used in adjuvant therapy for CRC treatment after surgery, often in regimens such as FOLFOX or XELOX. In advanced-stage (stage IV) patients, it is used in combination with targeted therapies such as cetuximab or bevacizumab [8]. For stage IV patients, the choice between combining targeted therapy with FOLFOX or FOLFIRI depends on individual responses. In cases of no response to irinotecan, the combination may be switched to oxaliplatin. However, there are no specific criteria for selection between these two drugs, and this decision is made at the discretion of the treating physician.

Precision medicine can significantly benefit from the ability to predict responsiveness to drugs, especially for stage IV patients, as it can allow prioritization of the drug that can be expected to deliver better efficacy. Presently, the conventional approach of precision medicine using patient-derived models such as patient-derived cells (PDCs) and patient-derived xenografts (PDXs) is being continued. However, PDCs have the limitation of exhibiting different features from the original human tumor, and PDXs face ethical approval issues, low success rates, and time-consuming processes. On the other hand, cancer patient-derived organoids (PDOs) offer the advantage of implementing an in vitro model similar to a patient’s cancer and showing a high success rate and efficiency [10,11]. Therefore, they are widely applied in precision medicine and are currently in use for precision medicine against CRC [12,13,14]. Hans Clever groups have implemented an in vitro drug-predictive screening model using PDO models. Although the drug-response predictions for irinotecan-based treatment have been successful, the correlation of oxaliplatin, a component of FOLFOX, to CRC remains unclear [15].

In this study, we investigated why oxaliplatin’s drug-response prediction using organoids is less effective and attempted to uncover the underlying reasons behind this phenomenon. Accordingly, we discovered that oxaliplatin resistance increases under conventional organoid culture conditions, the cause of which was identified as clusterin—an apoptosis inhibitor [16,17,18]. Based on this finding, a modified organoid medium condition was identified to enable accurate prediction by using a combination of culture compositions. The newly modified conditions were found to be successful in demonstrating the correlation between oxaliplatin and patient-derived CRC organoid drug response during drug screening. We aimed to improve the accuracy of drug response for FOLFIRI and FOLFOX, which are used in first-line treatment for CRC, using PDO models. Particularly, we developed novel modified medium conditions to implement a PDO prediction model for drug screening, which was not predicted previously.

As a result, we suggest a feasible and accurate PDO drug-response prediction model for CRC drugs. We believe that this research can contribute to increasing the survival rate of patients by improving the prediction of efficacy for first-line chemotherapy treatments for CRC. It can also contribute to the development of new drugs for CRC by using reliable PDO models instead of performing animal experiments or using 2D cell lines, which have their disadvantages.

## 2. Results

### 2.1. Oxaliplatin Resistance Is Induced by the Culture Conditions

**Figure 2:**
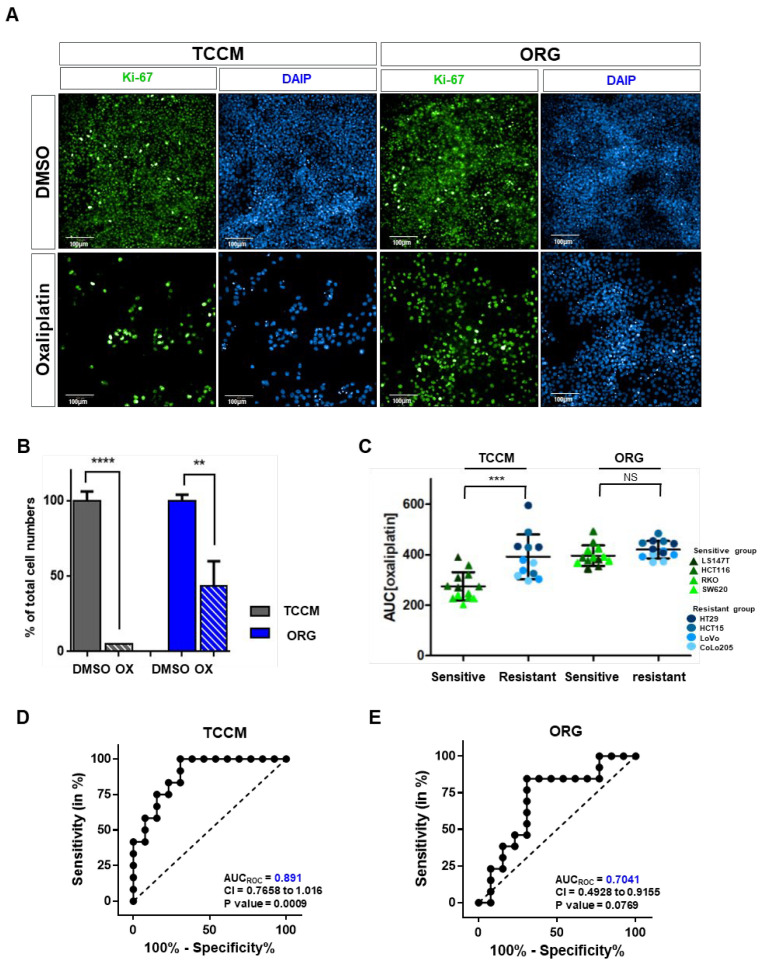
Comparison of reactivity between organoid and traditional culture conditions. (**A**) Representative images of immunofluorescence staining of Ki-67 and DAPI with either control and oxaliplatin for 2 h in TCCM and ORG media. Green = Ki-67, blue = DAPI. Scale bar = 100 μm. (**B**) Comparison of HCT116 cell numbers with and without oxaliplatin treatment under traditional cancer cell line culture conditions (TCCM; included 10% FBS) and organoid (ORG) culture conditions. Cell viability for each dose was normalized to control (DMSO vehicle) only cells. (**C**) Comparison of AUC with CRCs in two different culture media (TCCM/ORG). Triangles = sensitive CRC cell lines (LS147T, HCT116, RKO, and SW620 cells), dots = non-sensitive CRC cell lines (HT29, HCT15, LoVo, and CoLo205 cells). (**D**,**E**) The receiver operating characteristic (ROC) curve was plotted by the sensitivity (%) and 100-specificity (%) values for predicting the response rate. The data of (**C**) were plotted as an ROC curve. The experiment was conducted at least in triplicate. The dotted line represents an AUC_ROC_ of 0.5, which indicates no predictive value. CI, confidence interval. TCCM (**D**) and ORG (**C**) media. Values are presented as the mean ± SD. *p*-values: (**B**,**C**), two-tailed Student’s *t*-test. ** *p* ≤ 0.05; *** *p* ≤ 0.001; **** *p* ≤ 0.0001; NS: not significant.

Patient-derived organoids obtained from CRC specimens are a favorable model not only for drug development and biobank for diagnostic and therapeutic purposes but also for providing personalized precision medicine to patients (Supplementary Figure S1) [12,19]. This study highlights the difference between the drug responsiveness to commonly used first-line chemotherapy for CRC in vitro and clinical models. Therefore, we implemented a predictive model for drug responsiveness in vitro that closely resembles the clinical data. First, we observed the differential drug responsiveness of specific CRC cell lines in two distinct culture media (traditional cell line medium, TCCM; FBS 10% + P/S (100 U/mL) with Mccoy’s and Organoid medium, ORG; growth factors in advanced DMEM) (Figure 2A,B and Figure S2). Therefore, we cultured the representative CRC cell line (HCT116, ROK, SW6230, LS147T, HT29, HCT15, CoLo205, and LoVo), in both TCCM and ORG to examine their drug response. Finally, we divided them into two groups based on the half-maximal inhibitory concentration (IC_50_) value of 10 μM (sensitive group < 10 μM, resistant group > 10 μM) and the area under the curve (AUC) of the dose–response curve (DRC; sensitive group < 300, resistant group > 300). We selected HCT116 and HT29 as representative cells in the sensitive and non-sensitive groups (Supplementary Figure S3). We also analyzed the drug responses according to the culture media between the two groups [20,21]. The sensitive group displayed a significantly higher drug resistance in the ORG medium compared to the non-sensitive group. Next, we analyzed the AUC value for oxaliplatin between the two groups. In the non-sensitive group (HT29, HCT15, LoVo, and CoLo205 cells), there was no significant difference in the drug response with different culture media, but the drug response of the sensitive group (LS147T, HCT116, RKO, and SW620 cells) significantly differed with the culture media (Figure 2C).

In addition, when we drew the AUCroc curves of the sensitive group for each culture medium, we found that the TCCM showed a significant value of 0.891, but the drug response was difficult to predict in the ORG medium (Figure 2D,E). These experimental results demonstrated that the decrease in the predictability of drug response through the PDO model is related to the difference in the cell culture conditions. We hypothesized that this phenomenon is caused by an increase in drug resistance due to a media conditions during the drug screening process.

### 2.2. Oxaliplatin Response Is Attenuated by the Culture Conditions in a 2D/3D Model

**Figure 3:**
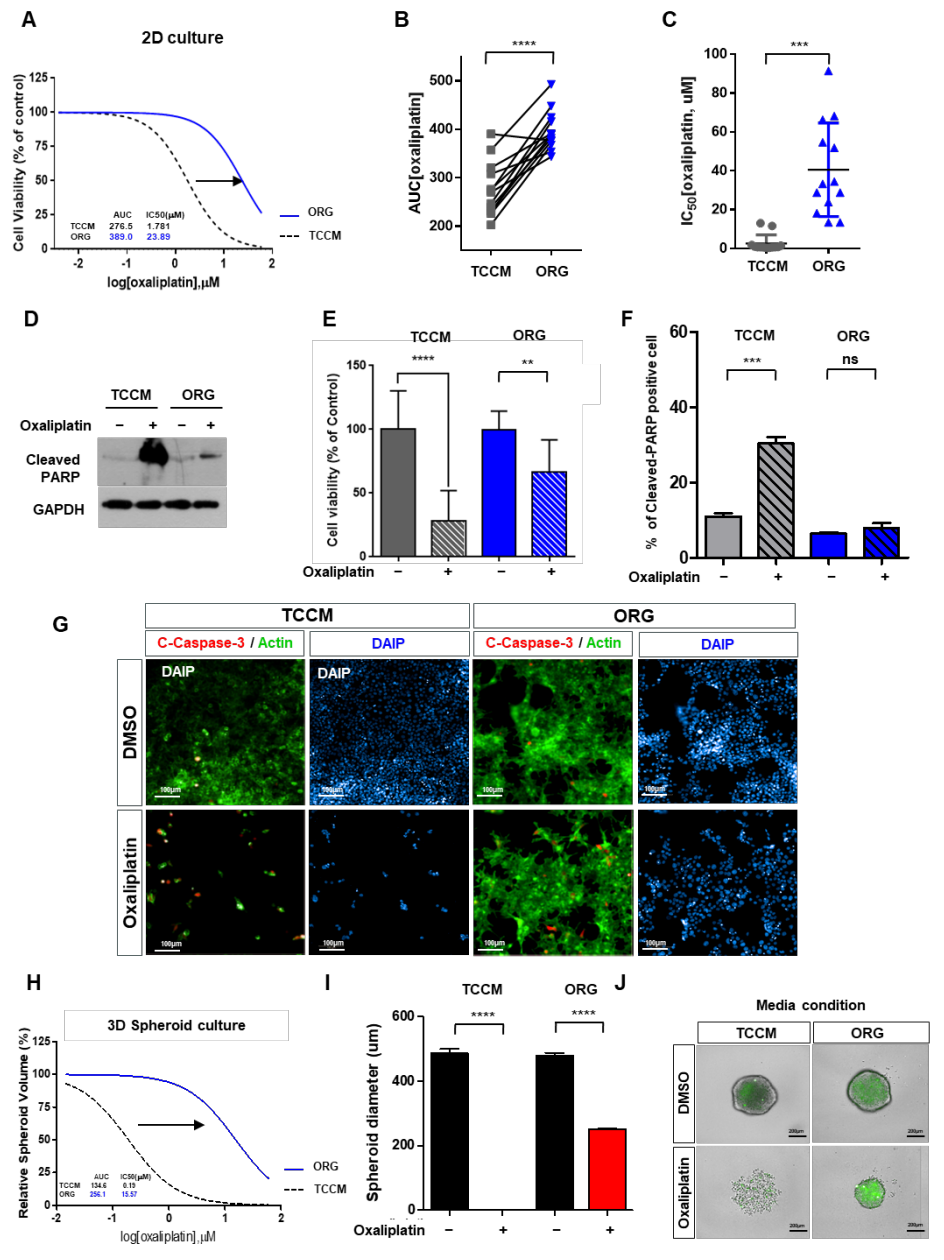
Comparison of sensitive group reactivity in traditional or organoid culture conditions. (**A**) HCT116 cells were administered an increasing dose of oxaliplatin (7 doses; 0.01464~60 μM) for 5 days. Cell viability was measured by ATPlite. Dose–response curve for HCT116 cells (oxaliplatin-sensitive cell) by oxaliplatin (OX) in TCCM and ORG media conditions. Cell viability for each dose was normalized to control (DMSO vehicle) only cells. Cell viability was measured by ATPlite. (**B**,**C**) Comparison of AUC (**B**) and IC_50_ (**C**) in the oxaliplatin-sensitive group (LS147T, HCT116, RKO, and SW620 cells) with two different culture media (TCCM/ORG). (**D**) Western blotting of cleaved PARP of HCT116 cells (oxaliplatin-sensitive cells) after treatment with 15 μM oxaliplatin for 24 h in two different culture media (TCCM/ORG). GAPDH was used as a loading control. (**E**) HCT116 cells were administered with 15 μM oxaliplatin for 5 days in different culture media (TCCM/ORG). Cell viability was measured by ATPlite. (**F**,**G**) The expression of apoptosis-related factors, cleaved PARP (**F**) and cleaved Caspase-3 (**G**), in response to oxaliplatin in TCCM and ORG media conditions by immunofluorescence analysis. Graph displaying the percentage of apoptotic HCT116 cells following treatment with oxaliplatin in TCCM or ORG (**F**). Representative images of immunofluorescence staining of cleaved caspase-3, actin, and DAPI with either control and oxaliplatin for 24 h in TCCM and ORG media. Red = cleaved caspase-3, green = actin, blue = DAPI. Scale bar = 100 μm (**G**). (**H**) Dose–response curve for HCT116−GFP spheroids (oxaliplatin-sensitive cell) with oxaliplatin (OX) treatments in TCCM and ORG media conditions. For tumor spheroid growth (3D culture), HCT116−GFP cells were cultured in 384-well ULA plates in a culture medium. The spheroid’s cytotoxicity was measured by HCS. Relative spheroid volume for each dose was normalized to control (DMSO vehicle) only cells. (**I**) GFP fluorescence signal of HCT116-GFP spheroids was acquired for 5 days following the drug treatments (oxaliplatin = 15 μM) in two different culture media (TCCM/ORG). Spheroids’ size was quantified at 5 days. Spheroids’ diameter = average of the width and length. Values are presented as the mean ± SD (n = 5). (**J**) Representative images of HCT116−GFP spheroids grown on ULA plates for 5 days following the drug treatments (OX) in two different culture media (TCCM/ORG). Scale bar = 200 μm. Values are presented as the mean ± SD. *p*-values: (**B**,**C**,**E**,**F**,**I**), two-tailed Student’s *t*-test. ** *p* ≤ 0.05; *** *p* ≤ 0.001; **** *p* ≤ 0.0001; ns: not significant.

To verify our hypothesis, we focused on examining the drug response, specifically within the sensitive group. We conducted a comparative analysis of cell viability using HTS (high-throughput screening) with equal doses of oxaliplatin treatment, which revealed a significant increase in drug resistance exclusively under the ORG medium (Figure 3A). Furthermore, we compared the AUC values of oxaliplatin treatments under the TCCM and ORG culture media conditions and found that the resistance substantially increased under the latter. Notably, the IC_50_ value under the ORG was approximately 20 times higher than that under the TCCM (Figure 3C).

To further investigate the drug resistance, we analyzed the results of Western blotting (Figure 3D) and cell viability assay (Figure 3E and Figure S4) and quantified the levels of cleaved PARP—an apoptosis-related protein (Figure 3F). The findings indicated similar amounts of cleaved PARP before and after oxaliplatin treatment under the ORG culture medium. Similarly, the expression of cleaved caspase 3, a well-known marker of apoptosis, displayed minimal changes in response to oxaliplatin in the ORG medium (Figure 3G). This finding led us to speculate that the ORG culture medium induced resistance to oxaliplatin by suppressing apoptosis under the 2D culture condition (Figure 3A–G).

When performing 3D cell culture experiments, the cell environment can be manipulated to mimic that of a cell in vivo and provide more accurate data about cell-to-cell interactions, tumor characteristics, drug discovery, metabolic profiling, stem cell research, and other types of diseases. To validate whether the observed pattern showed the same trend under 3D conditions, we cultured HCT116 spheroids on U-shaped ULA plates. In parallel, we treated HCT116 spheroids with oxaliplatin for 5 days and calculated the spheroid volume to assess viability (Figure 3H–J). Consistent with our previous findings, we observed exclusively elevated drug resistance under the ORG medium. The high-content screening (HCS) images of HCT116 spheroids treated with 15 μM oxaliplatin supported these results, thereby verifying increased resistance to oxaliplatin under the ORG medium (Figure 3J). Moreover, we investigated the drug response in different volume ranges based on the spheroid volume growth and compared the results. This analysis further confirmed the challenging nature of drug-response prediction with the increased oxaliplatin resistance in the ORG medium (Figure 3H–J). These findings together confirmed that not only in the 2D model but also in the 3D culture under the ORG medium, a significant decrease occurred in apoptosis with an accompanying increase in drug resistance.

### 2.3. Oxaliplatin Resistance Is Selectively Increased in Organoid Culture Medium

**Figure 4:**
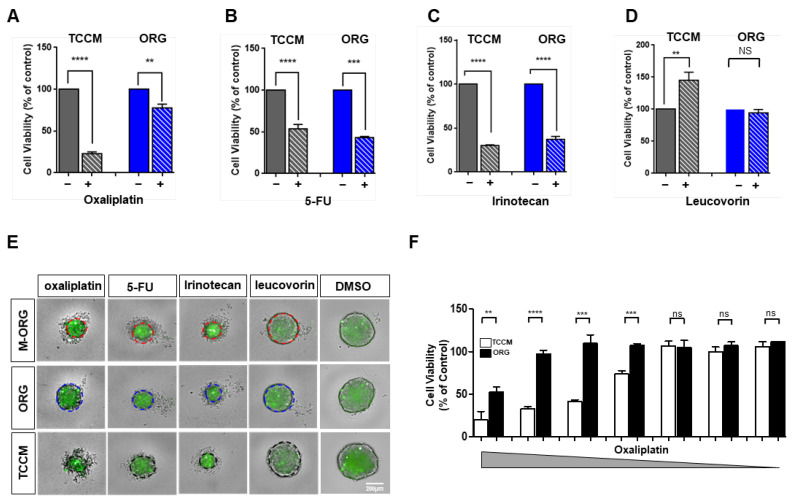
Oxaliplatin resistance selectively increases in the organoid culture condition. (**A**–**D**) HCT116 cells were administered with 15 μM oxaliplatin (**A**), 5-FU (**B**), irinotecan (**C**), and leucovorin (**D**) for 5 days in different culture media (TCCM/ORG). Cell viability was measured by ATPlite. Comparison of drugs included in first-line chemotherapy in terms of mean values of HCT116 cell viability in two different culture media (TCCM/ORG). First-line chemotherapy = FLOFIRI (irinotecan/5-FU/leucovorin), FOLFOX (oxaliplatin/5-FU/leucovorin). (**E**) Representative images of HCT116-GFP spheroids grown with oxaliplatin on ULA in three different culture media (TCCM/ORG/M-ORG). Scale bar = 200 μm. (**F**) HCT116 cells were administered a 7-point serial dilution series of oxaliplatin for 5 days in different culture media (TCCM/ORG). Cell viability was measured by ATPlite. Values are presented as the mean ± SD. *p*-values: (**A**–**D**,**F**), two-tailed Student’s *t*-test. ** *p* ≤ 0.05; *** *p* ≤ 0.001; **** *p* ≤ 0.0001; ns: not significant.

To authenticate the response of other drugs used in first-line chemotherapy for CRC, we treated HCT116 spheroids with oxaliplatin, 5-FU, irinotecan, and leucovorin at a concentration of 15 μM each, and obtained HCS images after 3 days. We observed a significant difference in the drug response in the case of oxaliplatin and leucovorin relative to that in TCCM (Figure 4A–E and Figure S5). In the case of leucovorin, no cell death was observed in the TCCM condition compared to the control group, but rather some cell growth was observed, and in the ORG condition, a similar response to the control group was observed. We analyzed cell apoptosis to compare all drugs used for first-line chemotherapy under different culture media. While other drugs displayed similar responses irrespective of the culture media, oxaliplatin demonstrated a rapidly increased resistance under the ORG medium (Figure 4F). In addition, on examining the response of HCT116 spheroids to oxaliplatin under different concentrations in both TCCM and ORG media, we noted that, in the ORG medium, the progression of cell death was repressed due to the increase in resistance at the same concentration. Similarly, when we analyzed this aspect using a bar graph, we did not find a significant difference in the drug response between the two culture media at relatively low concentrations, and the difference increased from 0.014~0.234 μM concentrations up to 0.9387~60 μM (Figure 4F).

### 2.4. Oxaliplatin Response Sensitivity Is Restored by Medium Modification

**Figure 5:**
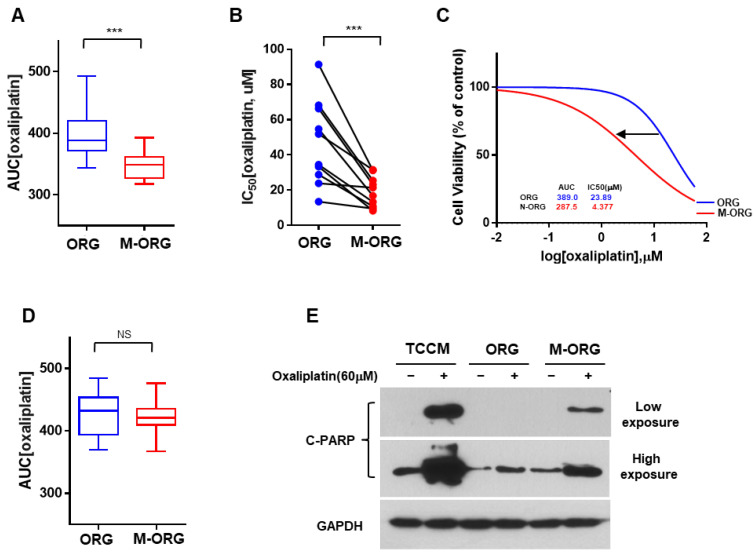
Determination of the modified condition to increase oxaliplatin’s sensitivity. (**A**,**B**) Comparison of AUC (**A**) and IC_50_ (**B**) in the oxaliplatin-sensitive group (LS147T, HCT116, RKO, and SW620 cells) with two different culture media (ORG/M-ORG). (**C**) HCT116 cells were administered an increasing dose of oxaliplatin for 5 days. Dose–response curve for HCT116 cells by oxaliplatin (OX) in ORG and M-ORG media conditions. Cell viability for each dose was normalized to control (DMSO vehicle) only cells. Values are presented as the mean ± SD (n = 5). (**D**) Comparison of AUC in the oxaliplatin-non-sensitive group (HT29, HCT15, LoVo, and CoLo205 cells) with two different culture media (ORG/M-ORG). (**E**) Western blotting of cleaved PARP of HCT116-GFP spheroids after treatment with 60 μM oxaliplatin for 24 h in two different culture media (ORG/M-ORG). GAPDH was used as the loading control. Values are presented as the mean ± SD. *p*-values: (**A**,**B**,**D**), two-tailed Student’s *t*-test. *** *p* ≤ 0.001; NS: not significant.

Previous studies have reported that the Wnt/β-catenin signaling pathway is associated with chemoresistance in CRC [22,23,24,25]. We hypothesized that Wnt/β-catenin-signaling-related factors in organoid cultures could induce oxaliplatin resistance. To corroborate the resistance of oxaliplatin to changes in media conditions, we used a combination of media compositions to prepare a novel culture condition, referred to as M-ORG (lacking Wnt/β-catenin signaling factors) (Supplementary Figure S2). We then compared the drug response of the CRC cell line to oxaliplatin in ORG and M-ORG media by analyzing the drug-response values (Figure 5A–D). We discovered that drug responsiveness increased in the M-ORG medium in the oxaliplatin-sensitive CRC group (Figure 5A–C). However, no change was observed in drug sensitivity in the oxaliplatin-non-sensitive group (Figure 5D).

To assess the extent of recovery of drug-response sensitivity, we determined the cleaved PARP levels (Figure 5E) and performed a cell viability assay (Figure 4E) of CRC spheroids in different media compositions (TCCM/ORG/M-ORG). We noted a similar pattern between TCCM and M-ORG with respect to the cleaved PARP levels with the newly modified medium. This observation indicated the recovery of drug-response sensitivity under the M-ORG medium condition (Figure 4E and Figure 5E), thereby demonstrating that oxaliplatin response sensitivity was restored by the modified medium.

### 2.5. Oxaliplatin Response Is Repressed by Clusterin under an Organoid Culture Medium

**Figure 6:**
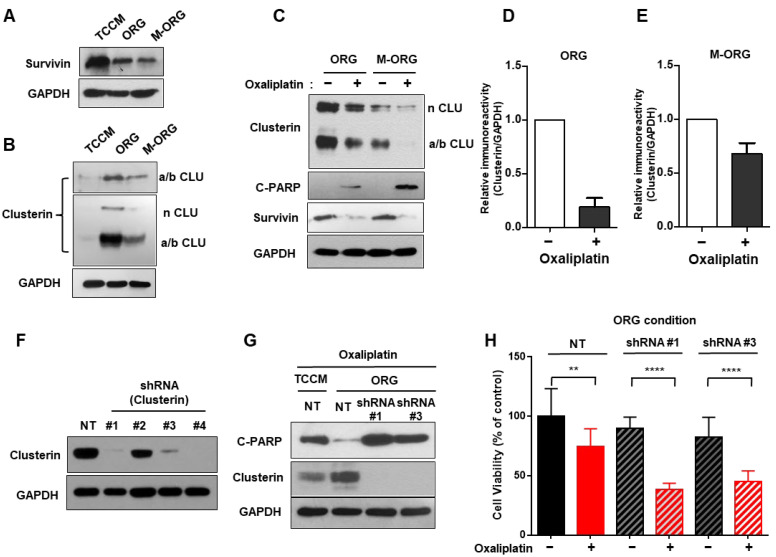
Oxaliplatin response is repressed by clusterin under an organoid culture medium. (**A**,**B**) Western blotting of survivin (**A**) and clusterin (**B**) of HCT116 cells for 24 h in three different culture media (TCCM/ORG/M-ORG). GAPDH was used as the loading control. (**C**) Western blotting of clusterin, survivin, and cleaved PARP of HCT116 cells after treatment with 15 μM oxaliplatin for 24 h in two different culture media (ORG/M-ORG). GAPDH was used as the loading control. (**D**,**E**) Densitometric analysis. Band intensities were normalized to GAPDH. Results are representative of three independent experiments. (**F**) Western blotting of the efficiencies of shRNAs knockdown of clusterin expression in infected HCT116 cells by shRNA lentivirus. GAPDH was used as the loading control. (**G**) Western blotting of cleaved PARP and clusterin in HCT116 cells that were transduced with control, clusterin shRNA. GAPDH was used as the loading control. (**H**) Cell viability assay of HCT116 cells from (**G**). Values are presented as the mean ± SD. *p*-values: (**H**), two-tailed Student’s *t*-test. ** *p* ≤ 0.05; **** *p* ≤ 0.0001.

In order to investigate the mechanism behind these changes, based on previous reports on oxaliplatin resistance [26], we examined survivin in response to drug treatment under M-ORG. We observed a decrease in survivin expression in ORG compared to TCCM, and there was no significant difference when comparing M-ORG to ORG. These results suggest that the changes in oxaliplatin drug sensitivity in ORG and M-ORG may not be attributed to survivin but could be due to other mechanisms (Figure 6A). On the contrary, clusterin displayed an increasing trend under the ORG medium. Accordingly, we speculated that a new mechanism may be involved. Based on the hypothesis that clusterin plays a role in apoptosis inhibition and cell survival, like survivin, we conducted the necessary experiments [16,17,18]. We next confirmed the reduction in survivin expression in the ORG media condition, but clusterin expression was increased in the ORG medium (Figure 6A,B). Moreover, when cultured in the M-ORG medium, the amount of clusterin protein was found to be reduced compared to that in ORG (Figure 6B–E).

To investigate the relationship between these protein expression changes and drug responsiveness, we treated organoids with oxaliplatin at a 15 μM concentration in each ORG and the M-ORG medium and conducted Western blotting. We observed that c-PARP expression was significantly higher in the oxaliplatin-treated M-ORG condition than in the ORG condition. Our results highlight a strong inverse correlation between clusterin and c-PARP protein levels, prompting the oxaliplatin resistance of clusterin in CRC. (Figure 6C).

To collect more direct evidence, we conducted experiments under the same conditions after knocking down clusterin in the same cells. We could confirm whether the cells were properly knocked down by determining the clusterin protein levels (Figure 6F). Next, we treated the cells with 15 μM of oxaliplatin. The results revealed high levels of clusterin and low levels of cleaved PARP, thereby indicating a correlation between clusterin and oxaliplatin resistance in ORG. Knocking down clusterin in ORG led to a decrease in the extent of chemoresistance. Notably, the cell’s clusterin knockdown exhibited higher levels of apoptosis when compared to the cells cultured in TCCM, which contains clusterin. These results clearly demonstrated the significant involvement of clusterin in oxaliplatin resistance (Figure 6G,H).

### 2.6. Patient-Derived Organoids (PDO) Can Predict Oxaliplatin Response in a Modified Medium

**Figure 7:**
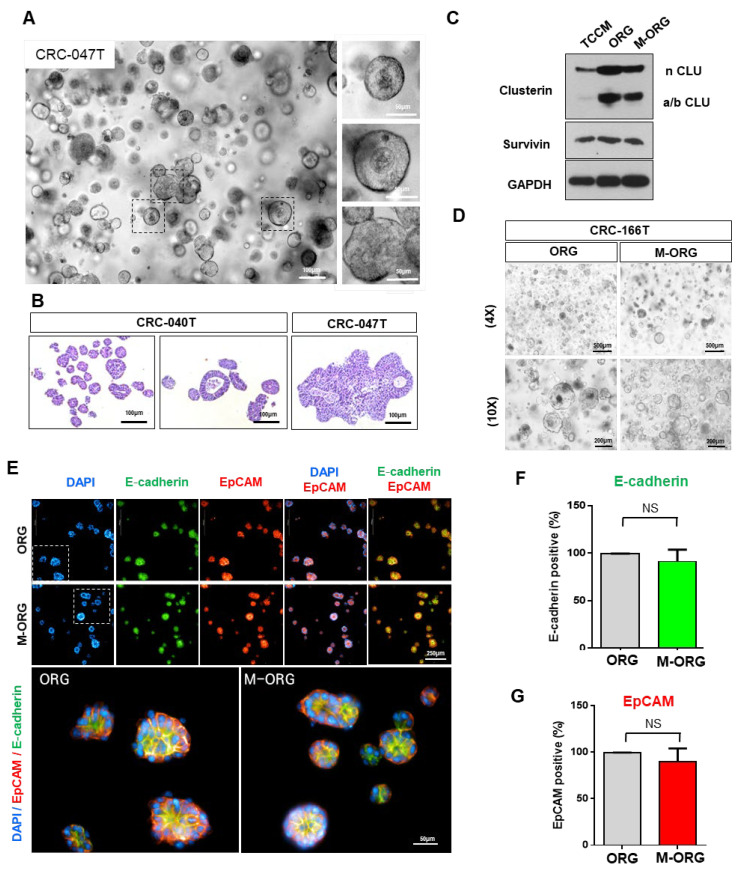
Characterization of CRC organoids under the newly modified medium. (**A**,**B**) All PDOs were cultured in ORG media conditions from CRC patient tumor tissues before drug responsiveness experiments. The organoid structure resembles the primary tumor epithelium. Most organoids have one or more lumens, resembling the tubular structures of a CRC tumor. Scale bar = 50 or 100 μm (**A**). Tumors with no lumen produce small organoids without a lumen. Hematoxylin–eosin (**E**) staining of formalin-fixed paraffin-embedded (FFPE) organoid sections revealed that tumor-derived organoids presented patient-specific heterogeneous morphologies. Scale bar = 100 μm (**B**). (**C**) Western blotting of clusterin and survivin of colorectal-cancer-patient-derived organoids (CRC-166T) for 24 h in three different culture media (TCCM/ORG/M-ORG). GAPDH was used as the loading control. (**D**–**G**) Comparison of CRC organoid characteristics between the ORG and M-ORG media. Representative images of CRC organoids in organoid(ORG) and modified (M-ORG) media, respectively. Scale bar = 100 μm (**D**). Representative immunofluorescence staining images display the protein expression level of EpCAM, E-cadherin, and DAPI in CRC organoids in two different culture media (ORG/M-ORG). Red = EpCAM, green = E-cadherin, blue = DAPI. Scale bar = 50 or 200 μm (**E**). (**F**,**G**) The bar graphs show the percentage positivity of (**F**) E-cadherin and (**G**) EpCAM staining in different culture media. Values are presented as the mean ± SD. *p*-values: (**F**,**G**), two-tailed Student’s *t*-test. NS: not significant.

To prove whether the tumor organoids from CRC patients maintain their organoid characterization in the M-ORG medium, we compared the traditional (ORG) and modified (M-ORG) organoid culture conditions. First, we cultured five types of CRC-patient-derived organoids under traditional (ORG) conditions and observed their histopathological features of POD (Figure 7A,B and Figure S6). We also observed changes in clusterin protein levels in CRC cell lines (HCT116) under various culture conditions (TCCM/ORG/M-ORG), as well as in colorectal-cancer-patient-derived organoids (CRC-166T). As shown in Figure 7D, similar morphologies were maintained under M-ORG conditions. In addition, we confirmed the expression of organoid markers such as E-cadherin and EpCAM through immunofluorescence analysis. CRC organoid markers displayed similar expressions under both the culture media (ORG and M-ORG). This finding suggests that the novel drug screening condition can replace drug screening using CRC-PDO. (Figure 7D–G).

### 2.7. CRC-PDO Drug Sensitivity Predicts Response to Treatment with Oxaliplatin in the M-ORG Media Condition

**Figure 8:**
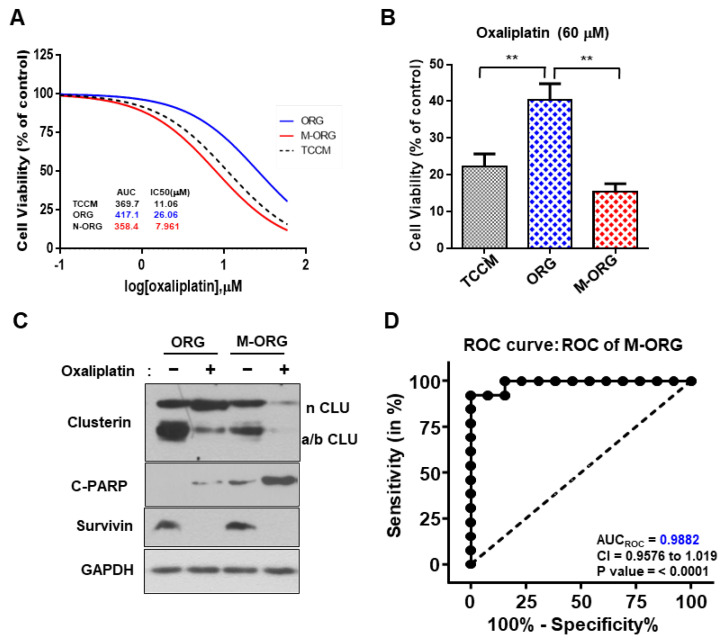
CRC PDO drug sensitivity predicts response to treatment with oxaliplatin in the M-ORG media condition. (**A**) CRC-166T organoids were administered increasing doses of oxaliplatin (0.024~100 μM) for 5 days in three different culture media (TCCM/ORG/M-ORG). Cell viability was measured by ATPlite. Cytotoxicity for each dose was normalized to control (DMSO vehicle) only cells. (**B**) CRC-166T organoids were administered 60 μM of oxaliplatin for 5 days in three different culture media (TCCM/ORG/M-ORG). (**C**) Western blotting of clusterin, survivin, and cleaved PARP of CRC-166T organoids after treatment with 60 μM oxaliplatin for 24 h in two different culture media (ORG/M-ORG). GAPDH was used as the loading control. (**D**) The receiver operating characteristic (ROC) curve was plotted by the sensitivity (%) and 100-specificity (%) values for predicting the response rate. The data in Figure 5A were plotted as an ROC curve. The dotted line represents an AUC_ROC_ of 0.5, which indicates no predictive value. CI, confidence interval. M-ORG media. Values are presented as the mean ± SD. *p*-values: (**B**), two-tailed Student’s *t*-test. ** *p* ≤ 0.05.

To confirm whether the conditions generated as described can be applied to the CRC-PDO model in use, we cultured CRC-166T organoids under the same three conditions (i.e., TCCM, ORG, and M-ORG) and treated them with oxaliplatin in the same range. CRC-166T patients were found to be clinically sensitive to oxaliplatin, which was confirmed in the PDX model (Supplementary Figure S7). Similarly, under the M-ORG condition, the resistance decreased when compared to that in the ORG and displayed a very similar response to the TCCM condition. The AUC and IC_50_ values were also specifically high in the ORG, which confirmed the increase in resistance, while both the values were found to be similar under the TCCM and M-ORG conditions (Figure 8A,B and Figure S8A). We also observed that c-PARP expression of CRC-166T was significantly higher in the oxaliplatin-treated M-ORG condition than in the ORG condition (Figure 8C and Figure S8B).

Thus, we confirmed that ORG media conditions could impair drug responsiveness during drug screening and that the M-ORG medium prepared by us in this study is an excellent new condition to correct this issue. The modified condition could improve the accuracy of the previously inaccurate oxaliplatin predictive drug screening model. In addition, the drug response curves exhibited approximately a 2-fold decrease in drug responsiveness when compared to that under the organoid culture condition. Moreover, when using this model for prediction, a feasibility study revealed higher specificity and sensitivity, with an AUCroc value of 0.9882, when compared to the existing model (Figure 8D). This study’s findings provide a solution to the hurdles faced previously by researchers, with significant implications for creating a patient-derived tumor organoid model for precision medicine with low accuracy.

## 3. Discussion

Several studies have been conducted on chemoresistance in CRC. According to the literature, the Wnt/β-catenin signaling pathway is associated with chemoresistance in CRC [22,23,24,25]. In particular, MMP7 has been found to regulate oxaliplatin by the Fas receptor, which is known as a cell apoptosis promoter [27,28,29]. Specifically, it has been suggested that molecules involved in regulating Wnt/β-catenin signaling, such as MMP7 and miR 506, are closely related to the efficacy of the first-line chemotherapeutic drug, oxaliplatin [29,30].

Based on these findings, a potential association is postulated between oxaliplatin resistance and the Wnt/β-catenin signaling pathway. In addition, past studies have demonstrated that cancer-associated fibroblasts (CAFs) are associated with drug-response sensitivity and that oxaliplatin resistance can induce epithelial-to-mesenchymal transition [31]. Furthermore, research has revealed that certain compositions, such as the presence of a ROCK inhibitor, in an organoid culture medium can impart anticancer effects in CRC [32,33].

Based on these previous studies, we hypothesized that the increased oxaliplatin resistance in CRC during drug screening is influenced by inhibitors or Wnt-related components present in the ORG culture medium. To test this hypothesis, we formulated a modified organoid medium by removing the relevant components and conducted drug screening by using the proposed modified medium “M-ORG”. The results of drug response evaluation with M-ORG suggested a decrease in resistance to oxaliplatin. Notably, this reduction in resistance was observed only in cell lines that were previously sensitive to oxaliplatin, while no change was observed in cell lines that were originally resistant. In particular, loss of function mutations in adenomatous polyposis coli (APC) is common in CRC, leading to inappropriate activation of typical Wnt signaling [34]. In this study, we mostly used ACP mutant CRC-PDOs (Supplementary Figure S6). Although conclusions need to be drawn based on our results using more CRC-PODs, it is unlikely that this will affect the growth of organoids under M-ORG conditions in the absence of Wnt-related components [35,36]. Based on these findings, we validated the hypothesis that activators and inhibitors present in an organoid culture medium contribute to oxaliplatin resistance in APC mutant CRC.

Previous studies have reported that the mechanism of oxaliplatin resistance is regulated by survivin/p38 [26,34,35]. However, in this study, we observed that expression of survivin, which has been reported to cause oxaliplatin resistance, actually decreased in the ORG condition compared to the TCCM condition. We found no significant changes in survivin expression under different culture conditions (ORG/M-ORG). Therefore, further to the research indicating the involvement of clusterin in the regulation of the Akt pathway and oxaliplatin response in hepatocellular carcinoma (HCC) [37], we conducted experiments to demonstrate the correlation between clusterin and oxaliplatin resistance. Through clusterin knock-out experiments, we verified that clusterin over-expression induced oxaliplatin resistance in ORG. Furthermore, as shown in Figure 6B and Figure 7C, clusterin expression, which was increased in ORG compared to TCCM, decreased in M-ORG, which should be due to clusterin-inducing oxaliplatin resistance changes.

The first-line chemotherapy for CRC is a critical decision that determines the overall survival (OS) [38,39]. Currently, there is no clear criterion for selecting the appropriate anticancer therapy, despite the presence of various research findings regarding the efficacy of first-line treatments. As a result, there is a growing demand for precision medicine to provide patient-individualized treatment [40,41]. It is important to maintain the characteristics of cancer organoids in our new culture condition, M-ORG, during drug screening to apply precision medicine. Accordingly, we conducted characterizations under specific culture conditions (such as TCCM/ORG/M-ORG) and detected no significant changes in the key biomarkers of cancer organoids. Thus, this study demonstrated the feasibility of using in vitro PDO models for precision medicine in APC mutant CRC.

However, for the presented M-ORG culture condition, this study needs to accumulate relevant clinical data by conducting drug screening using a larger number of PDOs and analyzing them comparatively. Particularly, incorporating various factors that influence the in vivo conditions, such as lifelog data and the microbiome, into these prediction algorithms can provide more accurate prediction models. With further research, our present findings can contribute to increased favorable prognoses and OS for CRC patients by providing personalized chemotherapy through high-precision prediction models.

## 4. Materials and Methods

### 4.1. Two-Dimensional Cell and 3D Spheroid Culture

For 2D cell culture, human CRC cell lines were purchased from ATCC. HCT116, HT29, and HCT15 cells were cultured in Mccoy’s medium supplemented with 10% fetal bovine serum (FBS; Thermo Fisher Scientific, Waltham, MA, USA) and penicillin/streptomycin (P/S; 100 U/mL; Thermo Fisher Scientific) at 37 °C under 5% CO_2_ conditions. RKO and LS147T cells were cultured in minimum Eagle’s essential medium (MEM; Gibco, Waltham, MA, USA) supplemented with 10% FBS and P/S (100 U/mL) at 37 °C under a 5% CO_2_ atmosphere. SW620 cells were cultured in RPMI 1640 supplemented with 10% FBS and P/S (100 U/mL) at 37 °C under 5% CO_2_ conditions. The LoVo cells were cultured in Kaighn’s modification of Ham’s F-12 medium (F-12k) supplemented with 10% FBS and P/S (100 U/mL) at 37 °C under a 5% CO_2_ atmosphere.

For the 3D spheroid culture, U-shaped, 384-well ultra-low attachment (ULA) plates (MS-9384UZ, S-bio, Hudson, NY, USA) were seeded with a suspension of 40 μL of the cell culture media containing 500 cells/well in triplicates per condition. The culture media used were medium-supplemented with 10% FBS and P/S (100 U/mL). For the drug screening, 500 cells were seeded in 20 μL media contained in a U-shaped 384-well ULA plate that was centrifuged at 200× *g* for 2 min. After 24 h of incubation, 2× concentration of the drug with 20 μL media was added into the wells.

### 4.2. CRC Specimen Processing and Derivate Organoid Culture

Fresh tumor tissue samples were processed as previously described in ref. [42], albeit with several modifications. After receiving the relevant informed consent, tumor specimens or malignant ascites with the corresponding clinical records were obtained from patients undergoing surgery or paracentesis at the Samsung Medical Center (SMC) in accordance with the associated Institutional Review Board (IRB file numbers 201004004 and 202109112). This work was performed in compliance with all relevant ethical regulations for research using human specimens. For organoid culture [15,42], the tissue was sliced into small pieces, rinsed in 70% ethanol and ice-cold PBS with P/S (300 U/mL) at least five times, and then minced in a 10 cm culture dish by using forceps and a blade or scissors. The minced tumor samples were resuspended in digestion-buffer DMEM (Gibco) containing P/S (100 U/mL), 2.5% FBS, collagenase type IV (75 U/mL; Gibco), and 125 μg/mL Dispase II (Gibco) and incubated for 20–30 min at 37 °C. Following digestion, the samples were pelleted and resuspended in fresh DMEM, passed through a cell strainer with a pore size of 100 μm (Falcon; Waltham, MA, USA), and centrifuged at 200× *g* for 3 min at 4 °C. Then, the pellet was resuspended in DMEM and recentrifuged at 200× *g* for 3 min at 4 °C to remove the debris and collagenase. The supernatant was collected and centrifuged at 200× *g* for 3 min at 4 °C. The cell pellet was then suspended with Matrigel (growth factor reduced; BD Biosciences, Franklin Lakes, NJ, USA) and dispensed into two 24-well culture plates (25 μL Matrigel/well). A BioMycoX^®^ Mycoplasma PCR Detection Kit (D-100; PELOBIOTECH GmbH, Am Klopferspitz, Germanay) was used to confirm the absence of mycoplasma.

### 4.3. Cell Viability and Proliferation Assay

HCT116 2500 cells were seeded in a 96-well plate. After 24 h of incubation, the cell medium was removed, and different condition media were added, (such as traditional cell culture medium (TCCM), organoid medium (ORG), and modified organoid medium (M-ORG)). The 96-well plates were incubated in a new culture medium for 24 h before treatment with 60 μM of oxaliplatin (S1224; Selleckchem, Houston, TX, USA). Staining and analysis were performed at 5 days post-drug-treatment to allow sufficient time for apoptosis following drug response. At 5 days, the cell medium was removed from the wells of adherent cells. Next, 100 μL of tetrazolium (0.2 mg/mL) salts was added to each well and incubated for 2 h at 37 °C in a 5% CO_2_ atmosphere before determining formazan crystal formation via microscopy. The supernatant was removed from the wells and formazan crystals were dissolved by the addition of 100 μL of isopropanol to each well in order to achieve homogenous optical density after 30 min. An additional column of empty wells received 100 μL of isopropanol to be read as blanks by a microplate reader. The absorbance of the purple-coloured product was quantified at 570 nm using a microplate reader.

### 4.4. Drug Screening

Drug screening was performed as described [43,44] previously. The CRC cell line and organoid, cultured in serum-free medium, were dissociated into single cells and seeded into a 384-well plate (500 cells/well) with technical triplicates. The CRC cell line and POD were treated with drugs in a four-fold and 7-point serial dilution series from 3.84 nM to 60 μM in TCCM, ORG, and M-ORG. After 5 days of incubation at 37 °C in a 5% CO_2_ humidified incubator, cell viability was determined using an adenosine triphosphate monitoring system based on firefly luciferase (ATPlite 1step; PerkinElmer, Waltham, MA, USA) and estimated by an EnVision Multilabel Reader (PerkinElmer). The relative cell viability for each dose was obtained via normalization with PBS per plate. DRCs were fitted using GraphPad Prism 6 (GraphPad Software Inc. San Diego, CA, USA). Best-fit lines and the resulting IC_50_ values were calculated using GraphPad: (log[inhibitor] versus normalized response). The equation for modeling log (inhibitor) vs. normalized response curve is Y = 100/(1 + 10^(X−LogIC50)^). The top and bottom are plateaued in the units of the *y*-axis and IC_50_ is the concentration of the drug that responds halfway between the bottom and top [44]. HillSlope was used to describe the steepness of the curves. The AUC for each DRC was calculated using the trapezoidal method, ignoring regions defined by fewer than two peaks. The AUC values from non-convergent or ambiguous DRCs were excluded from all analyses.

### 4.5. Plasmids and Lentiviral Transduction

Lentiviral vectors expressing shRNAs for clusterin and NT (non-target shRNA) were purchased from Sigma-Aldrich: clusterin (#1, TRCN0000078611; #2, TRCN00 00300844; #3, TRCN0000304143; #4, TRCN0000147204). For virus production, 293T cells were co-transfected with a lentiviral expression vector and packaging plasmids (psPAX2 and pCMV-VSV-G) using the Profection Mammalian Transfection System-Calcium Phosphate (Promega; Madison, WI, USA). Virus-containing supernatants were collected and concentrated by ultracentrifugation. The titer of each lentivirus was determined via serial dilution.

### 4.6. Antibodies

The following antibodies were used for immunoblotting: clusterin-α 1:200 (sc-5289; Santa Cruz, Santa Cruz, CA, USA), cleaved PARP (Asp124) 1:200 (5625; Cell Signaling, Danvers, MA, USA), survivin 1:1000 (sc-17779; Santa Cruz), GAPDH 1:5000 (sc-47724; Santa Cruz), and β-actin 1:1000 (sc-8432; Santa Cruz). The following antibodies were used for the immunofluorescence experiment: cleaved PARP (Asp124) 1:400 (5625; Cell Signaling), cleaved capsase 3 1:1600 (9664; Cell Signaling), Alexa Fluor™ 488 Phalloidin 1:400 (a12379, Invitrogen, Waltham, MA, USA), Hoechst 3342 1:2000 (H3570; Invitrogen), and Alexa Fluor^®^ 594 Goat anti-Rabbit IgG (H + L) Secondary Antibody 1:800 (A11012; Thermo Fisher Scientific).

### 4.7. Western Blot Assays

The experimental cells were lysed in Pierce^®^ IP lysis buffer (Thermo Fisher Scientific) supplemented with proteinase inhibitor and phosphatase inhibitor. After blocking, the blots were incubated with mouse monoclonal primary antibody overnight at 4 °C. After washing with TBS-T (1X Tris-buffered saline with 0.1% Tween^®^ 20 detergent), the blots were incubated with HRP-conjugated secondary antibody for 1 h at room temperature. Detection was performed using the SuperSignal^®^ West Pico Chemiluminescent Substrate (ECL, Thermo Fisher Scientific).

### 4.8. Immunofluorescence Analysis and HCS

For the immunofluorescence analysis, cells and tissues were fixed in 4% paraformaldehyde (PFA) in PBS for 20 min. After permeabilization and blocking with 0.3% Triton X-100 and 1% bovine serum albumin (BSA) in PBS, the cells were incubated with primary antibodies for overnight at 4 °C. The cells were washed three times with PBS, followed by incubation with diluted secondary fluorescent antibodies for 1 h. After final washing, cells were counterstained with PBS containing Hoechst 33258 and imaged using a × 20 objective in high-throughput imaging system (Operetta CLS; PerkinElmer). Image analysis and quantification was performed by dedicated imaging software (Harmony 4.6; PerkinElmer).

### 4.9. Statistical Analysis

All data are expressed as the means ± SD from at least three independent experiments. Quantification of immunopositive cells in immunostaining analyses was performed using NIH image J software (National Institutes of Health, Bethesda, MD, USA; http://rsb.info.nih.gov/nih-image/; accessed on 30 May 2023), and the results are presented as percentages of pixel mean area. For the animal survival studies, *p*-values were determined using a log-rank test. Student’s *t*-test was performed to determine the statistical significance. *p* < 0.05 was considered to indicate statistical significance.

## 5. Conclusions

We successfully established a novel drug screening model of CRC organoids for precision medicine. This study can serve as a foundation for the development of next-generation prediction models and can be used as training data for predictive modeling. If this research progresses, it can ultimately contribute to increased favorable prognoses and OS for colorectal cancer patients by providing personalized chemotherapy through high-precision prediction models.

## Data Availability

Research data that support the findings of this study are securely stored in an institutional repository and are available to share from the corresponding author upon reasonable request.

## Supplementary Materials

The following supporting information can be downloaded at: https://www.mdpi.com/article/10.3390/cancers15235531/s1, Figure S1: An overview of the drug-response prediction model for precision medicine, Figure S2: Components of cell and organoid drug screening media, Figure S3: Dose–response of oxaliplatin in CRC cell lines, Figure S4: Comparison of reactivity between organoid and traditional culture conditions, Figure S5: Oxaliplatin resistance selectively increases in organoid culture conditions, Figure S6: Clinical information of CRC patients and CRC organoids. Figure S7: Antitumor effect of oxaliplatin in the CRC(CRC166T) PDX model, Figure S8: Dose–response of oxaliplatin in CRC-PDO.
