# Supplementary material for: Improved Drug-Response Prediction Model of APC Mutant Colon Cancer Patient-Derived Organoids for Precision Medicine
Source: Cancers (Basel). 2023 Nov 22;15(23):5531. doi: 10.3390/cancers15235531 (PMC10705195; doi:10.3390/cancers15235531)
Supplement: Supplementary file 1 [file cancers-15-05531-s001.zip › cancers-2672231-supplementary.pdf]

Supplementary Figure S1. An overview of the drug-response prediction model for precision medicine.

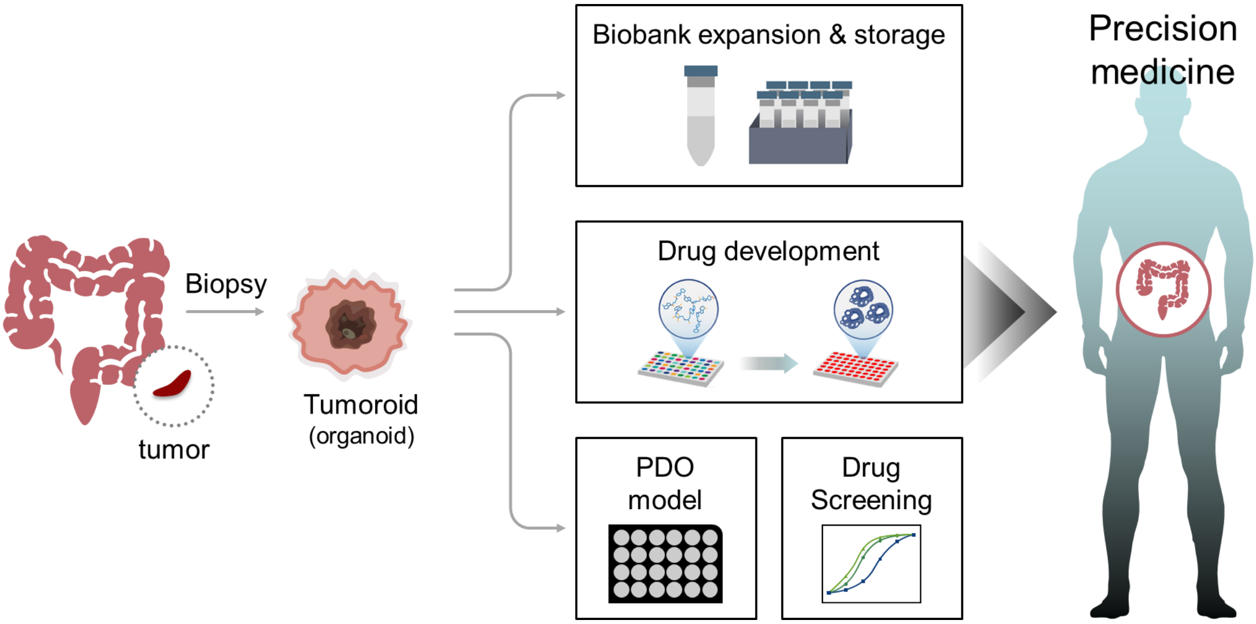

Supplementary Figure S2. Component of cell and organoid drug screening media

Component	Media condition		
	TCCM	ORG	M-ORG
McCoy's 5A Medium	+	-	-
FBS	+	-	-
Penicillin / Streptomycin	+	+	+
Advanced DMEM/F12	-	+	+
Glutamax	-	+	+
HEPES	-	+	+
Primocin	-	+	+
B27 supplement	-	+	+
N2 supplement	-	+	+
N-Acetylcysteine	-	+	+
Nicotinamide	-	+	+
Gastrin	-	+	+
EGF	-	+	+
A83-01	-	+	±
Y-27632	-	+	±
SB202190	-	+	±
Wnt3a	-	+	±
R-spondin 1	-	+	±
Noggin	-	+	±

Supplementary Figure S3. Dose response of oxaliplatin in CRC cell lines

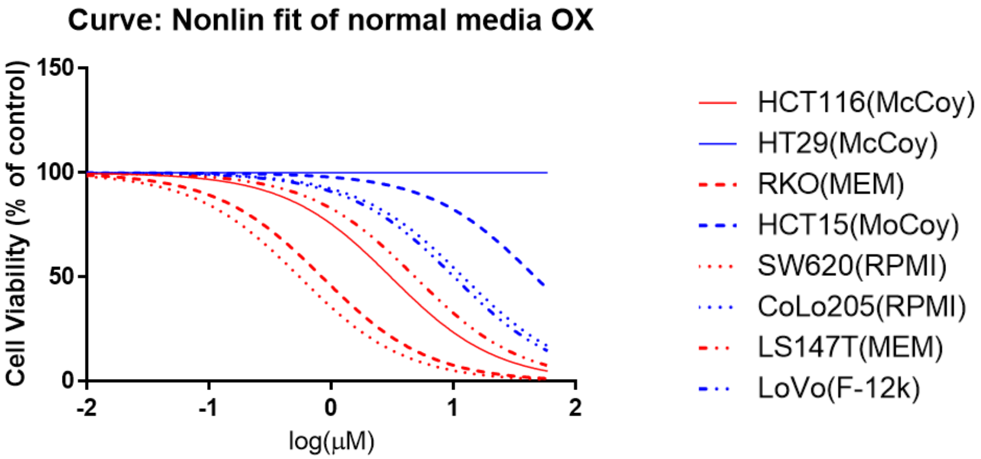

	HCT116(McCoy)	HT29(McCoy)	ROK(MEM)	HCT15(MoCoy)	SW620(RPMI)	CoLo205(RPMI)	LS147T(MEM)	LoVo(F-12k)
log(inhibitor) vs. normalized response								
AUC	296.0	513.0	234.7	414.9	222.6	327.0	326.6	340.9
LogIC50	0.4886	15.03	-0.07333	1.671	-0.2574	1.081	0.6878	1.007
IC50	3.080	1.073e+015	0.8446	46.93	0.5529	12.06	4.873	10.17

Supplementary figure. 3.

CRC cell lines were administered an increasing dose of oxaliplatin (7 doses; 0.01464–60 μM) for 5 days. Cell viability was measured by ATPlite.. Dose-response curve for CRC cell lines by oxaliplatin (OX) in TCCM media condition. Cell viability for each dose was normalized to control (DMSO vehicle) only cells. Cell viability was measured by ATPlite.

Supplementary Figure S4. Comparison of reactivity between organoid and traditional culture conditions.

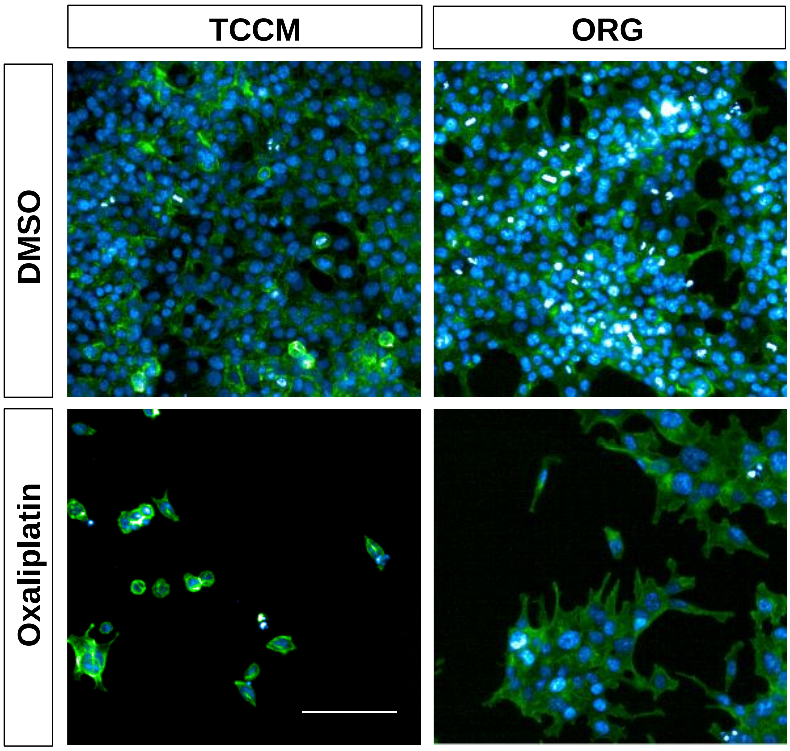

Supplementary figure. 4.

Comparison of HCT116 cell numbers in a traditional cancer cell line (TCCM, including 10% FBS), organoid (ORG) culture condition with a treat and non-treat oxaliplatin. Representative images of immunofluorescence staining of DAPI and actin in TCCM and ORG media with treated and non-treat oxaliplatin. Blue = DAPI, Green = F-actin. Scale bar = 100 μm

Supplementary Figure S5. Oxaliplatin resistance selectively increase in organoid culture condition

A

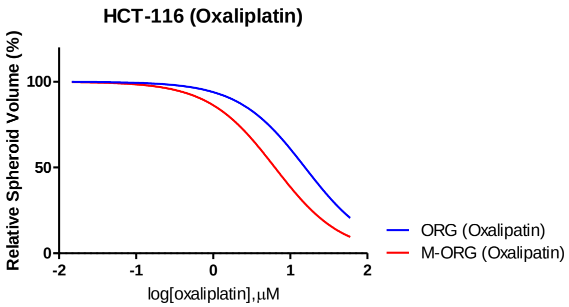

Oxaliplatin	ORG	M-ORG
IC_{50} (µM)	15.57	6.32
AUC	277.0	256.1

B

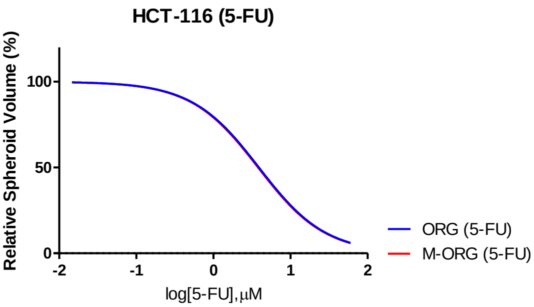

5-FU	ORG	M-ORG
IC_{50} (µM)	3.90	3.79
AUC	237.6	257.0

C

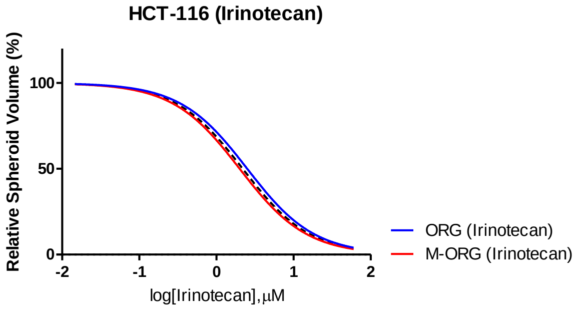

Irinotecan	ORG	M-ORG
IC_{50} (µM)	2.51	2.00
AUC	223.4	212.5

D

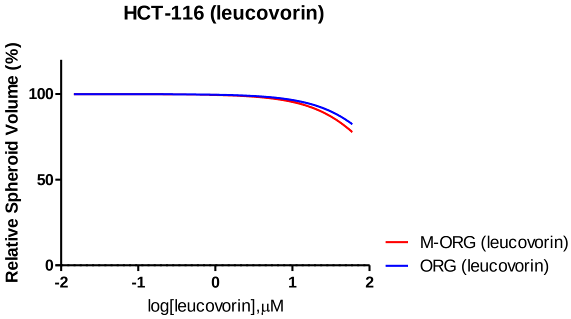

leucovorin	ORG	M-ORG
IC_{50} (µM)	281.0	210.4
AUC	315.0	358.8

Supplementary figure. 5.

(A-D) HCT116 cells (3D spheroid culture) were administered increasing doses(0.024–100 µM) of oxaliplatin (A), 5-FU (B), irinotecan (C), and leucovorin (D) for 5 days in three different culture media (TCCM / ORG / M-ORG). Cell viability of HCT 116 cells was measured by HCS (spheroid volume).

Supplementary Figure S6. Clinical information of CRC patients and CRC-Organoid

`		CRC patients			
	Sample No.	CRC-040T	CRC-047T	CRC-166T	CRC-237T
Clinical information	Sex	M	M	F	F
	Age	77	80	59	60
	Location	Sigmoid colon	Ascending	Sigmoid colon	Descending
	Stage	III	III	IV	IV
	Cell type	Well differentiated	Poorly differentiated	Moderately differentiated	Poorly differentiated
	MSI status	MSS[1)]	MSS[1)]	MSS[1)]	MSS[1)]
	1st Chemotherapy	No treatment	No treatment	XELOX[3)] , 8cycle	XELOX[3)] , 8cycle
	2nd Chemotherapy	-	-	-	XELIRI/Simvastatin
Mutation	KRAS	WT	p.G12D	p.G12S	p.G12D
	PIK3CA	WT	WT	WT	WT
	APC	p.E1295	p.Q195, p.E1317	p.T1475fs	p.V1804I
	CTNNB1	WT	WT	WT	p.R661Q
	TP53	WT	p.R174G	p.G113D	p.V11A

1) MSS : Microsatellite stable
2) FOLFOX : 5-FU/leucovorin/oxaliplatin
3) XELOX: Capecitabine and Oxaliplatin
4) XELIRI: Capecitabine and Irinotecan

Supplementary Figure S7. Antitumor effect of oxaliplatin in CRC(CRC166T) PDX model

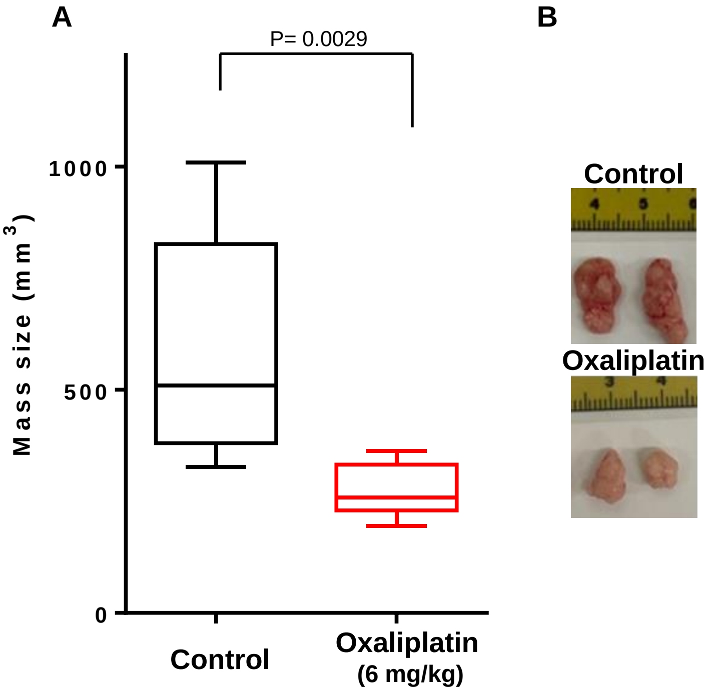

Supplementary Figure S8. Dose response of oxaliplatin in CRC-PDO

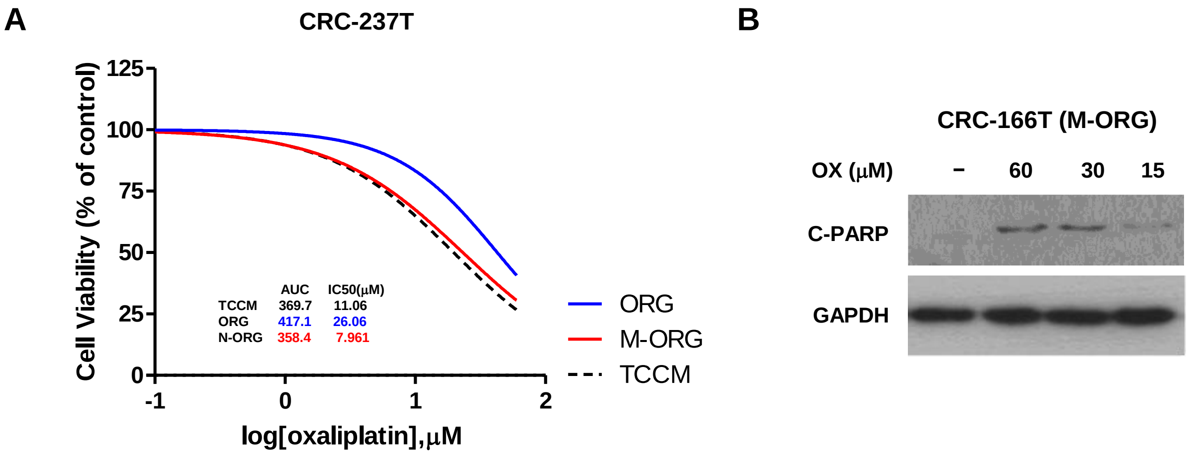

Supplementary figure. 8.

(A) Colorectal cancer patient-derived organoids (CRC-237T) were administered increasing doses of oxaliplatin (0.024–100 μM) for 5 days in three different culture media (TCCM / ORG / M-ORG). Cytotoxicity assay was measured by the high throughput screening (HTS). Dose-response curve was prepared for CRC-237T organoid by oxaliplatin in three different media (TCCM / ORG / M-ORG). Cytotoxicity for each dose was normalized to control (DMSO vehicle) only cells. (B) Western blotting of cleaved PARP(c-PARP) and GAPDH of Colorectal cancer patient-derived organoids (CRC-166T) after treatment with 60 μM oxaliplatin (OX) for 24 h in M-ORG culture media. GAPDH was used as the loading control.
